# Vancomycin-resistant *Enterococcus faecium* bacteremia in End-Stage Renal Disease (ESRD) and Focal Segmental Glomerulosclerosis (FSGS) patient under hemodialysis

**DOI:** 10.1016/j.heliyon.2024.e36028

**Published:** 2024-08-09

**Authors:** Mahrokh Rajaee behbahani, Farhad Moradi, Asiyeh Dezhkam, Reza Khashei

**Affiliations:** a Department of Bacteriology and Virology, School of Medicine, Shiraz University of Medical Sciences, Shiraz, Iran; b Department of Pediatric Infectious Diseases, Shiraz University of Medical Sciences, Shiraz, Iran; c Department of Microbiology, School of Medicine, Sabzevar University of Medical Sciences, Sabzevar, Iran

**Keywords:** *Enterococcus faecium*, Vancomycin-resistant, Bacteremia, Hospital infection, Case report

## Abstract

**Background:**

Today, one of the important challenges related to the emergence of antibiotic resistance among hospital-acquired infections is Vancomycin-Resistant Enterococci (VRE). The identification of the hospital transfer pattern and accurate laboratory diagnosis can be effective in preventing or selecting the appropriate antibiotics for the treatment of these types of infections, especially in hemodialysis patients.

**Case report:**

This report discusses the hospitalization of a 2.5-year-old boy with End-Stage Renal Disease (ESRD) and Focal Segmental Glomerulosclerosis (FSGS) at a nephrology center in a tertiary hospital. The patient received hemodialysis treatment, followed by an abdominal tap, which revealed an infection. Peritoneal and blood cultures were conducted using the BACT/ALERT®3D instrument, and the results indicated a bacterial infection during the hospital stay. Further analysis confirmed that the infection was caused by enterococci, and susceptibility testing revealed that the isolated strain was resistant to vancomycin. Fortunately, the enterococci infection responded well to linezolid antibiotic treatment, administered at a dose of 600 mg PO/IV q12hr for 14 days.

**Conclusions:**

This report highlights the importance of healthcare workers being aware of the transmission routes of VRE among patients. It emphasizes the need for appropriate hand hygiene, sterility of extracorporeal devices, and proper catheter care in medical centers.

## 1 Introduction

*Enterococcus* species as facultative anaerobic and gram-positive bacteria are part of the enteric microbiota. Although the pathogenesis of enterococcal infections is poorly understood, two enterococcal species, namely *Enterococcus faecium* and *Enterococcus faecalis* are the most common causes of human infections and isolated from different clinical specimens [1]. For example, they are responsible for the contamination of the indwelling catheters or equipment of the genitourinary tract that cause bacteremia, endocarditis, and urinary tract infections in hospitalized patients [1,2]. Besides, they can spread very quickly through person-to-person contact through the medical staff or contaminated medical equipment and supplies in hospitals, cause peritoneal or blood infections in hemodialysis patients [2]. Although broad-spectrum antibiotics such as vancomycin prescribed for the treatment of these microorganisms; today, vancomycin-resistant strains have been reported from dialysis patients and other patients with underlying diseases in different parts of the world [[1], [2], [3]]. Although one of the most important treatment challenges in the world is the treatment of Vancomycin-Resistant Enterococci (VRE), accurate laboratory identification and appropriate antibiotic prescription are very critical for effective therapy. Hence, we report a VRE case that was isolated from peritoneal and blood cultures of End-Stage Renal Disease (ESRD) and Focal Segmental Glomerulosclerosis (FSGS) patient with pleural effusion under hemodialysis who hospitalized in the nephrology center of a tertiary care hospital, southern Iran.

## 2 Case presentation

**Fig. 1:**
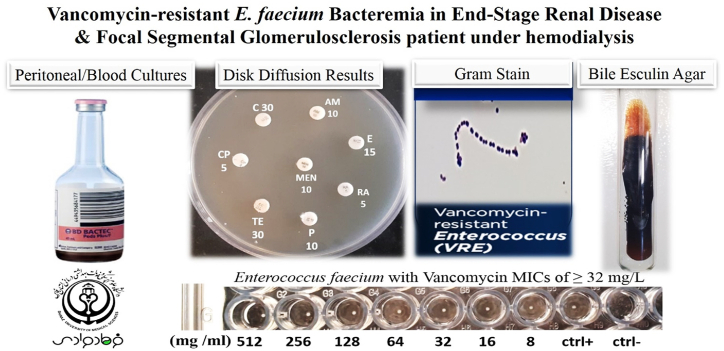
Phenotypic detection such as positive BACTEC bottle culture, gram-positive cocci in gram stain, well growing on bile esculin agar and antibiotics susceptibility test results for Vancomycin-resistant *E. faecium* bacteremia.

A 2.5-year-old boy with end-stage renal disease (ESRD) and familial focal segmental glomerulosclerosis (FSGS) presented with dysfunction of the peritoneal catheter, fever, and edema. Blood tests, urine tests, kidney ultrasound, and CT scan confirmed end-stage renal failure, while proteinuria, hyaline and broad waxy casts, histopathology findings, nephrotic syndrome, edema, fatigue, and loss of appetite indicated focal segmental glomerulosclerosis. A consultation with the general surgery department was conducted to evaluate the catheter's location, and subsequent catheter care was performed adequately. Additionally, an abdominal tap was performed, which revealed an infection. The ascites exam was completed one day after the onset of fever and peritoneal dialysis dysfunction. The initial tap of ascites revealed a total of 41,600 nucleated cells, with 96 % of them being polymorphonuclear cells (PMN). Subsequently, empirical treatment was started by prescribing intravenous cefepime. The initial laboratory results showed a white blood cell (WBC) count of 7.3 × 109 cells/L with 80 % neutrophils, a hemoglobin (Hb) level of 7.3 g/dL, a C-reactive protein (CRP) level of 113 mg/L, and an erythrocyte sedimentation rate (ESR) of 113 mm/h. Additionally, two consecutive peritoneal and blood cultures using the BACTEC system were positive for *Acinetobacter*. For this positive culture, empirically colistin (5000 unit/QD/Iv) was administered. However, successive BACTEC peritoneal and blood cultures results, after two weeks of hospitalization, showed enterococci. The contents of the BACTEC bottle were cultured in sheep blood agar medium and incubated overnight at 37 °C. Subsequently, non-hemolytic gray colonies were appeared after 24 h, and microbiological examination revealed gram-positive cocci arranged in chains in gram stain with catalase negative reaction, bacitracin and optochin resistant, and grew well on bile esculin agar. Indeed, during admission, the patient was taking dialysis peritoneal and because the cell counts of dialysis fluid had been increased, vancomycin was prescribed. Laboratory checkups for HIV Ab, HCV Ab, HBs Ag, *Mycobacterium tuberculosis*, and fungi demonstrated negative results. Although the catheter tip culture results showed heavy growth of enterococci, but the cell count of dialysis fluid had still been increased. It can be noted that during general surgery and hemodialysis for patients with underlying kidney diseases, especially for ESRD and FSGH cases, there is a risk of bloodstream infection with VRE. Moreover, Antibacterial Susceptibility Testing (AST) was performed by Kirby–Bauer disk diffusion method [4] according to Clinical and Laboratory Standard Institute (CLSI) 2021 guidelines [4]. According to the AST report, this isolate showed high resistance to tetracycline (30 μg), ciprofloxacin (5 μg), chloramphenicol (30 μg), meropenem (10 μg), ampicillin (10 μg), erythromycin (15 μg), penicillin (10 μg), and rifampin (5 μg). Furthermore, Minimal Inhibitory Concentration (MIC) for vancomycin was determined >256 μg/mL through the broth microdilution method according to CLSI 2021 guidelines [4]. Based on the AST results, it was found that VRE was susceptible to linezolid. Therefore, the antibiotic therapy was switched to linezolid (600 mg PO/IV q12hr for 14 days). Fig. 1 displays some of the main phenotypic detection tests and AST results. Totally, in this case two blood cultures and one culture from the tip of the catheter were collected, and all showed the presence of VRE. General surgery consult was done and the dialysis peritoneal catheter was disconnected. Thereafter, the patient did not take dialysis for 4–5 days. During admission, potassium (K) had been raised and symptomatic treatment was performed. After a few days, permcath inserted and hemodialysis started. Indeed, during the admission the patient showed high blood pressure and amlodipine was ordered. In our case, the *Acinetobacter* bacteremia was found to be susceptible to colistin, so polymyxin was prescribed as treatment. After undergoing polymyxin treatment, there was a decrease in the number of nucleated cells in the ascites fluid. On the third day, the cell count was 70 with 50 % PMN, on the fourth day, it was 100 with 9 % PMN, and on the fifth day, it was 80 % PMN with a cell count of 78 %. However, on the sixth day, there was a significant increase with a cell count of 1000 and 85 % PMN. Despite this rebound in cell count, the fever responded well to the polymyxin treatment, and the levels of CRP and ESR decreased from the first measurement (ESR was 113 mm/h and CRP was 113 mg/L). The peritoneal catheter remained in place. Due to the rebound in the number of nucleated cells in the ascites, we decided to repeat both the blood and ascites cultures. From the second culture, *Acinetobacter* was again detected. In the third culture, *Enterococcus* was isolated. Besides, Echocardiography findings showed Schwachman-Diamond Syndrome (SDS), good left ventricular (LV) systolic function, left aortic arch, no coarctation of the aorta (COA), and good right ventricular systolic function. Also, TTE and TEE were performed to rule out infective endocarditis following VRE bacteremia, and mild pleural effusion was reported. On the other hand, results disclosed no pulmonary hypertension, left ventricular hypertrophy, mild left pleural effusion, mild mitral valve regurgitation (MR), and mild right pleural effusion. Finally, pleural effusion (disorder) was mentioned as a conclusion. The patient was dialyzed 3 times in week with a low amount of heparin and an ultrafiltration rate of 500 mL/h/m2. Fortunately, our patient successfully treated by linezolid and discharged from the hospital. After discharge, tip of catheter was removed and permcath was inserted for hemodialysis. The source of the VRE infection was the catheter, and there was no connection between the infection, other patients in the same room, or medical equipment.

## 3 Discussion

The first case of vancomycin resistance was reported in 1988 [5]. According to different studies, VRE have introduced as an important nosocomial infection with endogenous and exogenous origin that cause uncommon infections among inpatients [[3], [4], [5]]. For instance, using broad-spectrum antibiotics make selective pressure that allows to these species with high antibiotic resistance potential to overcome microbiota, leads to long-term survival in hospital environments, and transmission between hospitalized patients through contaminated instruments or the hands of health staff [4,5]. Based on some reports, VRE cause cardiac surgical wound infections, native or prosthetic valve endocarditis [6], pleuropulmonary infection [7], superficial wound infection [[8], [9], [10]], blood infections in transplant recipients [11], and ventriculoperitoneal shunt infection [12]. In addition, among different hospitalized patients, patients undergoing dialysis appear to be at higher risk for VRE and other multidrug resistant bacteria [[13], [14], [15], [16], [17]]. There are two types of enterococcal vancomycin resistance. The first form is intrinsic resistance (*van* C) with low-level resistance (MIC with 2–16 μg/mL) and the second form is acquired through transmissible genetic elements (*van*A or *van*B) with high-level resistance (MIC ≥128 μg/mL) [18]. In two studies, VRE were isolated from ESRD patients during peritoneal dialysis [19,20]. These patients often had other underlying diseases. For example, they had diabetes mellitus and ESRD with infected dialysis graft [19], systemic lupus erythematosus with ESRD during hemodialysis [6], congenital cardiac anomalies with ESRD during hemodialysis [21] and uremic patient on hemodialysis [22]. In these patients, it is possible that hemodialysis catheters and underlying kidney diseases are the most important risk factors for methicillin-resistant *Staphylococcus aureus* and VRE bacteremia. To our knowledge, this is the first report of isolation of VRE from ESRD and FSGS patient with pleural effusion during hemodialysis in the nephrology center from south of Iran. Similar to our results, previous studies have been notified that *E. faecium* is typically multidrug resistant that exhibits a higher degree of antibiotic resistance to different antibiotics [23]. Vancomycin resistance was defined with MIC value of ≥32 μg/mL. Our results showed vancomycin MIC for this strain was >256 μg/mL. Although linezolid is considered as a main treatment for VRE infections through inhibiting bacterial protein synthesis; however, linezolid and vancomycin-resistant enterococci have been cited by some investigations [24]. Moreover, using broad-spectrum antibiotics such as vancomycin, host immune status, and disruption of the gastrointestinal mucosa are regarded as the possible risk factors for this type of resistance [24,25]. Similar to Babcock HM et al. report [21], our isolate was fortunately susceptible to linezolid and the patient successfully treated with it. In conclusion, VRE bacteremia in ESRD and FSGH patients during hemodialysis rarely reported. Health care workers, especially in the neonatal or pediatric intensive care units, should be conscious and learn about transmission routes of VRE among hospital personnel and patients. Also, during general surgery and hemodialysis, appropriate hand hygiene, sterility of the extracorporeal devices, and catheter care must be considered especially patients with underlying diseases such as renal failure, immunocompromised and infants’ cases. Appropriate infection control measurements and prevention of the spread of this infectious agent in hospitals and medical centers are indispensable. Moreover, the prevalence of these antibiotic-resistant bacteria should be periodically followed-up, especially in the dialysis departments and operating rooms according to the hospital infection quality control programs.

## Funding

This work did not receive any specific grant IDs.

## Informed consent

In this report, written informed consent for publication of this study and accompanying images was obtained from the patient.

## Data availability statement

Data included in article/supp. Material/referenced in article. No additional information is availablefor this paper.

## CRediT authorship contribution statement

**Mahrokh Rajaee behbahani:** Formal analysis, Data curation, Conceptualization. **Farhad Moradi:** Writing – review & editing, Writing – original draft, Methodology, Investigation, Formal analysis, Data curation, Supervision, Validation. **Asiyeh Dezhkam:** Supervision, Methodology, Investigation, Conceptualization. **Reza Khashei:** Writing – review & editing, Conceptualization, Validation, Supervision.

## Declaration of competing interest

The authors declare that they have no known competing financial interests or personal relationships that could have appeared to influence the work reported in this paper.
